# Silk fibroin carriers with sustained release capacity for treating neurological diseases

**DOI:** 10.3389/fphar.2023.1117542

**Published:** 2023-05-05

**Authors:** Xinqi Huang, Yumei An, Shengye Yuan, Chen Chen, Haiyan Shan, Mingyang Zhang

**Affiliations:** ^1^ Institute of Forensic Sciences, Suzhou Medical College, Soochow University, Suzhou, China; ^2^ Department of Orthopedics, Dongtai People’s Hospital, Dongtai, China; ^3^ Department of Obstetrics and Gynecology, The Affiliated Suzhou Hospital of Nanjing Medical University, Suzhou, China

**Keywords:** silk fibroin, sustained release, neurological diseases, TBI, traumatic brain injury, drug delivery

## Abstract

Neurological diseases such as traumatic brain injury, cerebral ischemia, Parkinson’s, and Alzheimer’s disease usually occur in the central and peripheral nervous system and result in nervous dysfunction, such as cognitive impairment and motor dysfunction. Long-term clinical intervention is necessary for neurological diseases where neural stem cell transplantation has made substantial progress. However, many risks remain for cell therapy, such as puncture bleeding, postoperative infection, low transplantation success rate, and tumor formation. Sustained drug delivery, which aims to maintain the desired steady-state drug concentrations in plasma or local injection sites, is considered as a feasible option to help overcome side effects and improve the therapeutic efficiency of drugs on neurological diseases. Natural polymers such as silk fibroin have excellent biocompatibility, which can be prepared for various end-use material formats, such as microsphere, gel, coating/film, scaffold/conduit, microneedle, and enables the dynamic release of loaded drugs to achieve a desired therapeutic response. Sustained-release drug delivery systems are based on the mechanism of diffusion and degradation by altering the structures of silk fibroin and drugs, factors, and cells, which can induce nerve recovery and restore the function of the nervous system in a slow and persistent manner. Based on these desirable properties of silk fibroin as a carrier with sustained-release capacity, this paper discusses the role of various forms of silk fibroin-based drug delivery materials in treating neurological diseases in recent years.

## 1 Introduction

**FIGURE 1:**
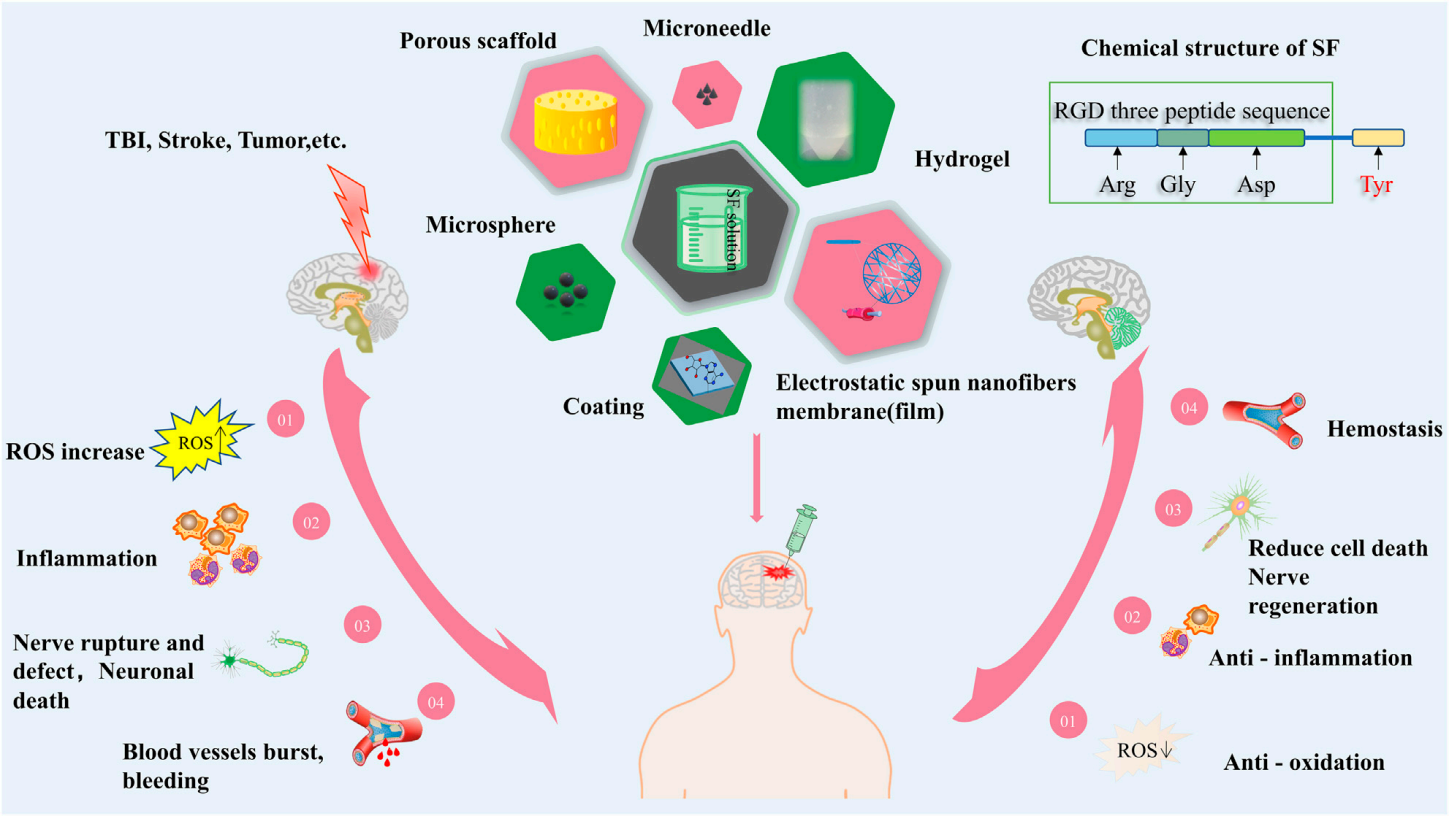
The molecular mechanism for functionalized SF materials to treat brain diseases.

**FIGURE 2:**
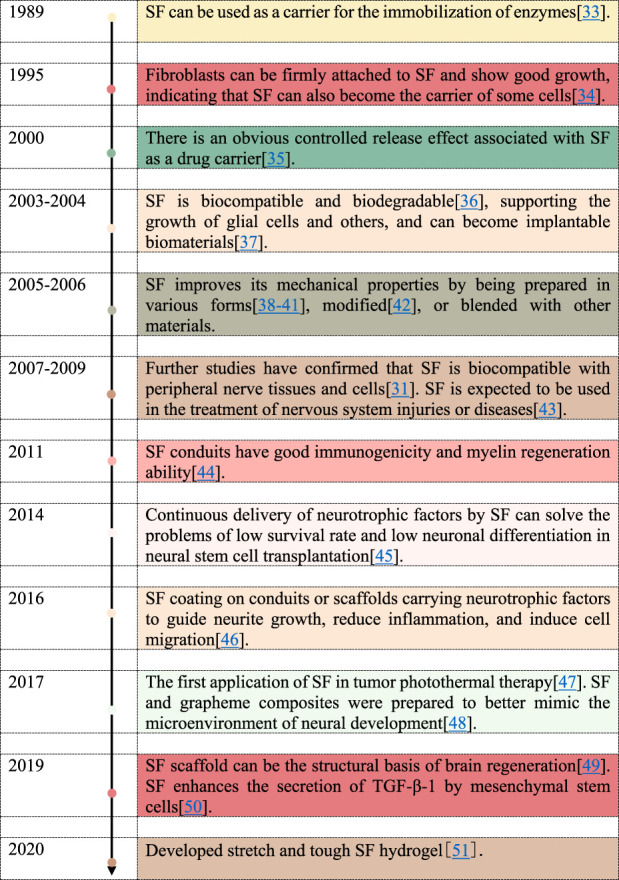
Timeline of SF as a drug carrier in neurological disease.

Neurological diseases, both acute and chronic neurological diseases, result in a loss of neuronal function due to the damage of neurons and neuronal networks, which may cause permanent disability such as cognitive impairment (Balcom et al., 2021). Therefore, appropriate treatment is critical for neurological diseases. Innovative and effective therapeutic strategies are urgently required to repair injured neural tissue. Stem cell-based therapy is becoming increasingly popular and has been used in a variety of neurological diseases, such as traumatic brain injury (Mahmood et al., 2004), Parkinson’s disease (Bouchez et al., 2008), spinal cord injury (Abrams et al., 2009), amyotrophic lateral sclerosis (Forostyak et al., 2011), and multiple sclerosis diseases (Yan et al., 2018). However, the low efficiency and high cost of the delivery system limited its practical application. Gene therapy shows promising superiority in rare genetic diseases but often suffers from irreversible adverse reactions (Morris and Schorge, 2022). A recent study also has found that regeneration and repair of nerve tissue can be stimulated by biomaterials (Lim and Spector, 2017; Shan et al., 2019; Yonesi et al., 2021). Moreover, nano-and-micron technologies also offer the possibility of biomaterial-cell interactions (Orive et al., 2009). Biomaterials can not only help transplanted cells survive and incorporate into damaged regions but also enable sustained drug delivery on a site-by-site basis. Therefore, biomaterials can improve drug delivery to the brain and promote neuronal regeneration and repair in combination with cell therapies (Elliott Donaghue et al., 2014). Biomaterials can be classified into natural or synthetic according to the origin and the components, such as extracellular matrix (ECM) (Song et al., 2018). In contrast to natural biomaterials, synthetic materials lack cell binding domains, resulting in weak bioactivity toward cells *in vivo* (Troy et al., 2021). The natural biomaterials commonly used in nerve regeneration are polyesters, proteins, and polysaccharides, such as silk fibroin (SF), alginate, hyaluronic acid, collagen, chitosan, and gelatin (Fornasari et al., 2020). SF has a long history of application as a drug carrier in different systems. The capability of purification, biocompatibility, and excellent binding capacity for a wide range of drugs of SF-based natural biomaterials makes it attractive for use as drug delivery carriers (Farokhi et al., 2020). But the use of SF as a drug carrier in neurological diseases has not been reported. Based on the advantages mentioned above, SF is expected to become a drug carrier for central nervous system diseases. However, it is different in the characteristics of central nervous system diseases, and different diseases require the application of different forms of SF to achieve the delivery of drugs and achieve the purpose of treating diseases. In our group’s research, SF hydrogels are effective in regenerating brain tissue, repairing damaged brain tissue, and improving neurologic recovery (Chen et al., 2022a; Zhang et al., 2022; Yang et al., 2022). Particular attention is being paid to the use of hydrogels in drug delivery due to their unique physical properties. As a result of their porosity, drugs can be loaded into the hydrogel matrix and released at a rate dependent on the diffusion coefficient between the small molecules or macromolecules through the gel matrix (Hoare and Kohane, 2008). There are also porous 3D structures made out of SF, including films, sponges, foams, and scaffolds that are useful for biomedical applications such as tissue engineering and implantable medical devices. Methods of use include filling, application, and injection. SF films were well tolerated in the brain without acute neuroinflammation, cell death, or altered brain function (Tang-Schomer et al., 2019). As a drug carrier and cell culture substrate, SF films were used to simulate the *in vivo* interface between drug reservoirs and brain cells for drug delivery in the brain (Tang-Schomer et al., 2019). Small molecules released from the SF membrane can penetrate the hippocampal region and remain in the ipsilateral hemisphere (Tang-Schomer et al., 2019). A variety of processing methods are available to tune the morphological and molecular mechanisms for functionalized SF materials as drug carriers to treat brain disease as follows (Figure 1). Dhyani et al. developed a UV-based strategy to modify SF nanogels and encapsulate bioactive molecules, which could be released at different rates. The bioactive molecules exhibited various delivery behaviors at specific regions to achieve the desired multiple biological effects (Dhyani and Singh, 2017). The carriers derived from the regenerated SF show high universality for small molecule drugs (adenosine (Wilz et al., 2008)), proteins (enzymes (Wang et al., 2007a), antibodies (Guziewicz et al., 2011)), antitumor drugs (Yucel et al., 2014; Montalbán et al., 2018), antibacterial drugs (Pritchard et al., 2013; Zhong et al., 2015), neurotrophic factors and cells (mesenchymal stem cells (Martín-Martín et al., 2019), sciatic nerve Schwann cells (Yang et al., 2007; Gu et al., 2014), neurons, etc.). Therefore, SF as a drug carrier is a suitable platform to achieve the treatment of neurological diseases. The timeline of SF as the drug carrier in neurological disease as follows (Figure 2). However, the research on SF as a drug delivery system for neurological diseases is still limited, such as how to strengthen the biological characteristics of SF itself, change the morphology of SF, and the effect of drug-loading to meet the needs of specific neurological diseases. In this review, we will focus on the advantages of different forms and applications (filling, application, and injection) of SF, and drugs delivered by SF for the treatment of neurological diseases, as well as the latest applications (including treatment and detection) in a few neurological diseases (including hemorrhagic and ischemic stroke, Alzheimer’s disease and Parkinson’s, traumatic brain trauma, glioblastoma and neuroblastoma, epilepsy, etc.).

When the nervous system suffered from injured, the body experiences several changes, including a higher level of reactive oxygen species (ROS), an increase in inflammation, ruptured blood vessels, nerve damage or rupture, and neuron death. Cells, growth factors, and chemical drugs can be carried into the body to repair damage by various forms of SF. A) The hemostatic ability of SF can be stimulated by the cross-linking of Tyr under the action of the inducer. B) The RGD sequence of SF (Arg-Gly-Asp) is conducive to cell adhesion, so SF can ensure the transport and adhesion of neural stem cells to the site of injury, further proliferation, and differentiation to repair the injured nerve. C) Although SF itself has a little anti-inflammatory effect, it can be combined with other antibacterial materials to form new biomaterials, which can inhibit or kill microorganisms and reduce cell death.

## 2 Different forms of silk fibroin carrier for drug delivery

A major aim of advanced drug carrier is to deliver the drug to the desired area and thereby reduce the systemic dose to decrease side effects (Farokhi et al., 2020). SF carrier was first reported in 1989 and a series of studies revealed the feasibility of SF carriers to load cells, enzymes, and small molecules. The material parameters of SF and the drug release behavior of SF hydrogels can be thoroughly investigated by experimental rheological methods, coupled models of affinity-based diffusion, degradation, and mechanical behavior. The result showed that SF had good mechanical properties and the ability to slow release and control release. Based on these properties, the drug release characteristics could be predicted, and the drug dosage could be adjusted in time (Gharehnazifam et al., 2021). The mechanical properties of the electrospinning SF mats were improved after wetting, which was more suitable to be used as drug carrier (Phamornnak et al., 2022). The electrospinning SF mat has good physical properties and can be used as neural substrates to promote the growth of neurons (Nune et al., 2019). In conclusion, various forms of SF scaffolds have different rheological and mechanical characteristics and are suitable for the treatment of different neurological diseases (Li et al., 2016; Montalbán et al., 2018; Cui et al., 2021). Continuous research indicated that various drugs could be regulated for sustained release by changing the state, conformations, and nanostructures of SF and blending with other materials, which implies the advantages of SF as an ideal smart carrier to better simulate the microenvironment *in vivo* (Mathur and Gupta, 2010; Wang et al., 2016a; Kapoor and Kundu, 2016; Farokhi et al., 2018; Gou et al., 2019; Yavuz et al., 2019; Wang et al., 2020; Horo et al., 2021). SF has multiple features that are beneficial to nerve regeneration. SF microspheres, gels, scaffolds, catheters, and other forms (Rockwood et al., 2011) were prepared to encapsulate stem cells (Ciocci et al., 2018), neurons (Zhang et al., 2012; White et al., 2015), and growth factors (Uebersax et al., 2007) to clarify the role and advantages of SF in the recovery of central and peripheral nervous systems.

### 2.1 SF microspheres

Polymer microspheres with the size of 1–500 μm have been used widely to encapsulate drugs. SF microspheres of different sizes have changeable penetrability, which can deliver drugs and active factors to different lesion sites in the human body. Compared with synthetic polymeric microspheres, biodegradable SF microspheres have several unique advantages in the delivery of various drugs, including proteins, nucleic acids, polypeptides, and other bio-macromolecules. Wang et al. evaluated the delivery of growth factors from polylactic co-glycolic acid (PLGA) and SF microspheres on human bone marrow-derived mesenchymal stem cells (hMSCs)-derived osteochondral differentiation. The result showed better osteogenic differentiation of hMSCs in the SF microsphere system because SF microspheres showed quicker sustained release of growth factors than PLGA microspheres. However, the fabrication process of SF microspheres is complicated (Wang et al., 2009). Gong et al. developed a polyethylene glycol (PEG) assisted emulsification method to encapsulate octreotide acetate with SF microspheres and octreotide could release slowly from SF microspheres for more than 28 days in rats after intramuscular injection (Gong et al., 2019). Shen et al. prepared BMP-2 encapsulated with SF microspheres in SF/nHAp porous scaffolds, which showed a sustained release of BMP-2 for 3 weeks (Shen et al., 2016). In another study, the composite alginate hydrogels combined with SF microspheres could provide a more sustained release of IGF-1 than microspheres without SF (Feng et al., 2020). The SF microspheres have a promising future as long-acting controlled release delivery. Hino et al. reported that the secondary structure of SF microspheres obtained by spray-drying changed from a β-sheet to a random structure. Due to water absorption and subsequent recrystallization, its conformation changes into β-sheet again. The β-folded structure is the most stable structure in the secondary structure of SF, which may affect its sustained release performance. The deficiency of the spray drying method is that the conformations conversion to the β-folded structure in SF microspheres may be obstructed if the particles are dried too fast. Lammel et al. produced SF particles by salting SF solution with potassium phosphate. Compared with emulsification, salt leaching voids the use of organic solvents and is friendly and safe. However, the whole experiment condition is in the environment of PH = 8, and the particles will gather into non-dispersible aggregates with poor pelletizing if PH = 5, suggesting that the change of PH value will affect the aggregation of SF microspheres (Lammel et al., 2010). Therefore, the specific production process for SF microspheres limited its practical application in biomedicine to a certain extent.

### 2.2 Silk fibroin hydrogels

With unique biocompatibility and biodegradability, SF hydrogels are one of the effective candidate materials for biomedical engineering, with many properties such as capacity to hold water, stability of shape and modifications to the functionality possibility. Consequently, a three-dimensional microenvironment that mimics extracellular matrix can be created, allowing cellular behavior and tissue function to be regulated. In addition to physical and chemical hydrogels, the SF hydrogels make promising biopolymeric matrices that are capable of controlling drug release (Tomeh et al., 2019). Wu et al. incubated the diluted silk solution at 60°C for 24 h to make it self-assemble to form a physical hydrogel and then dissolved doxorubicin (DOX) directly into the hydrogel to form hydrogel-loaded DOX. DOX can be released in and out of the body for more than 4 weeks and tumors treated with DOX-loaded filamentous hydrogels showed a larger necrotic area than those treated with free DOX (Wu et al., 2016). Lovett et al. mixed SF with bevacizumab solution to induce gelation by ultrasound and the SF hydrogels achieve drug-release for more than 3 months (Lovett et al., 2015). Attention should be paid that the influences, such as an increase in local temperatures, an engineering shear, and gas-liquid interface increase, affected the gelation of SF during the ultrasound process.

Chen et al. mixed SF hydrogels with carboxymethyl chitosan (CMCS) into SF/CMCS hydrogels, which can be easily controlled in terms of thickness, quality, and shape by electrodeposition on graphite (Chen et al., 2021). Guziewicz et al. developed a lyophilized SF hydrogel, which makes SF hydrogels suitable for long-term sustained release applications in antibody release (Guziewicz et al., 2011). In terms of physical strength and stability, hydrogels made by chemicals are highly effective compared with physical hydrogels. The common preparation methods for chemical hydrogels include photopolymerization, chemical crosslinking agent, and irradiation. He et al. found that tannic acid-reinforced methacrylated chitosan/methacrylated SF hydrogels (TA-CSMA/SFMA) had a significant effect on wound healing in a full-thickness skin defect model (He et al., 2020). It should be noted that the addition of a photoinducer may cause adverse reactions, such as surface oxidation. Kim et al. optimized bone tissue engineering using SF composite hydrogels with HAP nanoparticles (NPs) by irradiating SF composite hydrogels with HAP nanoparticles (NPs) and these hydrogels enhanced the osteogenic differentiation of hMSCs *in vitro* (Kim et al., 2017). Zhou et al. prepared porous polyvinyl alcohol/SF/nano-hydroxyapatite (PVA/SF/N-HA) composite hydrogels by genipin (GP) crosslinking and the composite hydrogels could be a novel approach to corneal tissue engineering (Zhou et al., 2019). Therefore, both physical and chemical cross-linking can induce gelation of SF solution, and the corresponding SF gels loaded with drugs can be prepared with the conditions optimized using improved methodology according to the performance of loaded drugs and experimental purposes. In biomedical applications, SF hydrogels are limited by their poor mechanical properties and swelling behavior (Yang et al., 2022). The biological safety of SF hydrogels and their composite hydrogel materials, hydrogels’ drug loading efficiency, and the excellent injection performance of hydrogels is important challenges in the field of regenerative medicine (Liu et al., 2022a).

### 2.3 Silk fibroin coating/film

SF coating can be obtained by dipping coating and layer-by-layer assembly. The application of SF coating can improve the “ initial burst release” of drugs and prolong the release time of drugs. Meanwhile, the behavior of drug-release can be precisely controlled by adjusting the thickness and crystallinity of SF coating. Pritchard et al. coated adenosine tablets with SF solution and put them into phosphate buffer solution (PBS) after methanol treatment for *in vitro* release. They found that the increase of the crystal content or the thickness of SF coating could deliver adenosine for several weeks and then degrade in the body, requiring no surgical removal (Pritchard et al., 2010). Wang et al. prepared SF coating by a step deposition process and used it as a carrier to incorporate drugs and the release duration of these adulterated molecules can be prolonged by controlling the coating structure and suppressing the initial burst (Wang et al., 2007b).

SF membrane can be obtained by solution pouring and freeze-drying. Lee et al. investigated antithrombogenicity and surface properties of SF and S-carboxymethyl keratin (SCMK) polymers by solvent cast, which show better antithrombotic properties than SF or SCMK alone (Lee et al., 1998). Seib et al. poured the SF solution onto the substrate and dried it to generate a SF membrane with different crystallinity. After different steam temperatures, dried SF membranes were immersed in doxorubicin solution and then were implanted in the corresponding tumor sites in breast tumor mice. The result showed that the different crystallinity of SF membrane produced at different temperatures provided a controlled release of doxorubicin and limited adverse reactions in the treatment of human breast cancer (Seib and Kaplan, 2012). The result suggests these systems as a good candidate material for SF coating or film to control drug release, when considering the all-aqueous process involved, coatings with conformal properties, and the degradability and biocompatibility of SF coating or film. A number of drawbacks, however, include expensive coatings, which together reduce affordability and commercial viability, and prevention of direct contact between loaded drugs and cells by coatings to the observation that insufficient manifestation of their effects may occur (Kwon et al., 2021).

### 2.4 Silk fibroin scaffold/conduit

SF porous scaffolds have well-interconnected pores and can well simulate the three-dimensional network environment in organisms (Moisenovich et al., 2019), to facilitate cell adhesion, proliferation, and growth (Abdel-Naby et al., 2017). The preparation methods of SF porous scaffold include freeze-drying, salt leaching, and porogen leaching. A frozen-dried SF/hyaluronan scaffold was prepared, and 3 weeks of mesenchymal stem cell culture were performed on the scaffolds by Garcia-Fuentes et al. In comparison to plain SF scaffolds, stem cell cultures on SF/hyaluronan scaffolds produced more effective tissue formation (Garcia-Fuentes et al., 2009). A salt leaching method was used to prepare demineralized bone matrix-SF (DBM/SF) scaffolds. The scaffolds can promote cell attachment and growth, and it may be a suitable candidate material for bone tissue engineering (Ding et al., 2015). However, traditional porous scaffolds generated by porogen can hardly control the distribution of scaffolds’ pores. Ze et al. combined supercritical carbon dioxide (SC-CO2) technology with porogen leaching using ammonium bicarbonate (AB) particles as a pore-forming agent, which could overcome the disadvantages of traditional porogen leaching and prepared scaffold was loaded with Schwann cells to demonstrate its good cytocompatibility (Li et al., 2019). Zhang et al. prepared bioactive glass (MBG)/SF scaffolds with aspirin to repair calvarial defects in the mice model. The MBG/SF scaffolds have been shown to enhance drug loading *in vitro*, increase drug release rate, and stimulate bone regeneration *in vivo* (Zhang et al., 2014).

SF conduits are more widely used in the preparation of nerve catheters (NC). The use of artificial nerve conduits (NCs) can be useful in the treatment of damaged peripheral nerves in certain cases, however, their therapeutic effect is often not enough effective (Madduri and Gander, 2012). Therefore, it is necessary to manufacture NCs to achieve a better therapeutic effect. Studies have shown that SF NCs promote axon regeneration in the short nerve gap thanks to the slow degradation of SFs (Wang et al., 2008; Cao and Wang, 2009). Schwann cells, in particular, can survive, adhere, and migrate along silk fibers due to their affinity for them (Yang et al., 2007). SF conduits are produced in three major ways: mold casting, staining, and electrospinning. Yang et al. used stainless steel casting molds by lyophilized demoulding to provide SF-NC and SF vessels that are implanted into the back of adult rabbits could degrade rapidly and meet the requirements of peripheral nerve regeneration compared with SF fibers (Yang et al., 2009). Ghaznavi et al. used catheterization immersed in SF/polyethylene oxide to examine the cellular inflammatory response and functional recovery in models of sciatic nerve defects and the catheter had physical and biological properties of promoting nerve repair from proximal to distal nerve stump (Ghaznavi et al., 2011). Wang et al. combined SF with PLGA to prepare nerve guidance conduits (NGCs), which showed the strength of good biocompatibility, stiffness, and high porosity (Wang et al., 2015). In addition to the traditional methods mentioned above, Carvalho et al. developed an SF-based cross-linking catheter for the delivery of neurotrophic factors. Studies have shown that this design has significantly improved retrograde transport, neuronal protection, and motor nerve reinnervation, and to some extent solved the defect of incomplete nerve regeneration caused by a lack of neurotrophic factors in the proximal region (Carvalho et al., 2021). You et al. fabricated a multi-channel bioactive SF nanofiber catheter, which can promote neuron-like development and directional neurite extension *in vitro* and *in vivo* spinal cord injury models (You et al., 2020). Despite its advantages, the scaffold can also be disadvantageous. It is difficult to distribute cells evenly and remain underpopulated in the central or core regions (Luetchford et al., 2020). The scaffold design relies on a bottom-up approach that may spread cells evenly and shape them into the final 3D structure by layering different cells or drugs (Chen et al., 2011). Likewise, simple hollow SF NGCs cannot enhance functional and sensory recovery after peripheral nerve injuries (PNIs). It has been shown that including neurotrophic factors (NTFs) in NGCs can significantly improve nerve regeneration (Carvalho et al., 2021). It is important, therefore, to find optimal delivery methods for drugs such as neurotrophic factors (NTFs) that are extracted from SF conduits.

### 2.5 Silk fibroin microparticles/nanoparticles

It has been reported that nanoparticles are effective carriers for drugs that promote efficacy. It shows unique characteristics due to the “size effect”. The common methods for preparing SF nanoparticles include desolvation, salt leaching, and supercritical fluid technologies. Kundu et al. prepared SF nanoparticles loaded with VEGF using a desolvation technique using dimethyl sulphoxide (DMSO) as the desolvation solvent. *In vitro* release experiments showed that there was no sudden release of VEGF in the drug delivery system, which could be used as an efficient release system of VEGF (Kundu et al., 2010). Lammel et al. produced SF particles by salting the SF solution with potassium phosphate. Compared with emulsification, salt leaching avoids the use of organic solvents and is friendly and safe. The whole experimental condition is in the environment of PH = 8. Increasing SF concentration will lead to larger particles. When PH = 5, particles will gather into non-dispersible aggregates with poor spheronization (Lammel et al., 2010). The preparation of SF nanoparticles was carried out by solution-enhanced dispersion by supercritical CO_2_ (SEDS). The SEDS process was used to encapsulate the non-steroidal anti-inflammatory drug Indomethacin (IDMC) in SF nanoparticles. The experimental results showed that SF nanoparticles prepared by the SEDS process were suitable for drug delivery as a biocompatible carrier and feasible for drug delivery control (Zhao et al., 2012). There is a great potential for SF microparticles/nanoparticles to be used as delivery systems for a wide variety of therapeutic agents, such as small molecules, protein drugs, genes, and vaccines, which enhances the biological stability and improves the drug delivery (Pham and Tiyaboonchai, 2020). The unspecific targeting of SF microparticles and nanoparticles may result in low therapeutic efficiency and systemic toxicity, due to their inefficiency as drug delivery systems.

### 2.6 Silk fibroin microneedle

In order to deliver localized treatment, microneedles (MNs) were used to penetrate the layers of the cuticle, which can achieve subcutaneous injections and continuously releases the drug directly into interstitial fluid or blood vessels by adjusting the swelling properties of MNs. The production method of SF microneedles is relatively simple and usually cast by mold. Qi et al. poured proline/melatonin/SF mixture into a polydimethylsiloxane microneedle mold to obtain drug-loaded SF microneedles and microneedles incorporated the drug into the body through the skin and maintained a high concentration of melatonin in the blood of rat insomnia model (Qi et al., 2021). Yavuz et al. reported that levonorgestrel was loaded into microparticles and then injected into silk-based microneedles, which could achieve continuous release for up to a year for unwanted pregnancy (Yavuz et al., 2020). Microneedle devices can also provide highly reproducible results and therapeutic benefits by penetrating through the cuticle into the interstitial fluid. However, there are some limitations to microneedles in biomedical applications, such as skin irritation or allergy to sensitive skin and broken microneedle tips left in the skin (Waghule et al., 2019).

In addition to the above-mentioned forms of SF carriers for drug delivery, Hu et al. used the electrostatic spinning method of preparation of the regenerated SF nanofibers and cultivated Schwann cells on SF nanofibers. They found that the regeneration of electrostatic spinning nanofibers simulates the structure and function of extracellular matrix form and the adhesion, growth, and proliferation of Schwann cells, showing excellent biocompatibility (Hu et al., 2012). In addition, studies have explored that straining flow spinning (SFS) can be used to regenerate high-performance silk fibers as scaffolds for axonal growth induction and guidance and spontaneous cellular connection of dissociated neurons (Mercado et al., 2020). Zhu et al. prepared a porous scaffold called SF sponge to grow primary cortical cells (with or without microglia removal). The results showed that neuronal maturation and synaptic development were reduced in fibroin sponges depleted by microglia (Zhu et al., 2020). Overall, a variety of SF forms, as biomaterial carriers, can be generated in a variety of applications using the method described. We summarized the advantages and disadvantages of different forms of SF carriers for drug delivery (Table 1).

**TABLE 1:** The advantages and disadvantages of SF carrier for drug delivery.

Forms	The preparation methods	Drugs	Application	Route of medication	Advantages	Disadvantages	References
Microspheres	Liposome template method	rhBMP-2 rhIGF-I	osteochondral differentiation		Drug release is fast and uniform. Degradation of products did not affect drug activity	The operation is tedious and repeated freezing thawing, and subsequent processing are needed	Wang et al. (2009)
	Liquid drying method	Octreotide	the treatment of acromegaly and gastrointestinal tumors	Intramuscular injection (suspended in carboxymethyl cellulose solution CMC)	Increased loading; prolonged release time	Poor uniformity	Gong et al. (2019)
	Microfluidic method	IGF-1	MI (myocardial infarction)	Intramuscular injection (hydrogel made from IGF-1 and alginate)	Long sustained release time	Organic solvents need to be added	Feng et al. (2020)
	Spray drying	Theophylline			After absorbing water (tissue fluid, etc.), the conformation changes to a stable β fold	It is not suitable for protein polypeptide drugs with poor thermal stability	Hino et al. (2003)
Gels	Self-assembly	Adriamycin	Breast cancer	Subcutaneous implant	Good thixotropy Release in and out of the body for at least 4 weeks The operation is simple	Time-consuming	Wu et al. (2016)
	Ultrasonic	Bevacizumab	Age-related macular degeneration	Intravitreal injection	Stable structures are formed at the nanoscale	Local temperature increase Mechanical shearing Gas-liquid interface increases	Kim et al. (2010), Lovett et al. (2015)
	Electric field effect	Carboxymethyl chitosan (CMCS)	Heal skin wounds	Covering skin wound	Reduce manufacturing costs Reducing the risk of high-pressure shocks Retter equipment stability		Chen et al. (2021)
	Lyophilization	protein therapeutics (Monoclonal antibody)			high protein stability significant stabilizing effect long-term storage		Guziewicz et al. (2011)
	PH adjustment (CO_2_)	Sericin	Heal skin wounds	Covering skin wound	Clean	The addition of foreign polymers is required to improve vulnerability and poor flexibility. Acidic PH is not conducive to cell survival	Napavichayanun et al. (2019)
	Organic solvent (ethanol)	Hydroxyapatite	Bone Tissue Engineering		Hydrogel is formed quickly		Ribeiro et al. (2015)
	photopolymerization	Tannins	Heal skin wounds	Covering skin wound	Water resistance Strong mechanical properties	An inducer may cause an adverse reaction	He et al. (2020)
	Irradiation	Hydroxyapatite	Bone Tissue Engineering		Hydrogel is formed quickly	Radiation	Kim et al. (2017)
	Chemical crosslinking agent	Hydroxyapatite	Corneal Tissue Engineering			Toxic	
Coating	Dip coating	Adenosine		Implantation	Delay drug release time		Pritchard et al. (2010)
	Layer-by-Layer assembly	Rhodamine B and Even Blue			Delay drug release time		Wang et al. (2007b)
Film	Solution casting	S-carboxymethyl keratin			The antithrombotic		Lee et al. (1998)
	Freeze drying	Adriamycin	Breast tumor	Local implanted	Controlling temperature (crystallinity) can control drug release		Seib and Kaplan (2012)
Porous scaffolds	Lyophilization	Mesenchymal stem cells			Simplicity of operator	The size of the bracket aperture fluctuates greatly	Garcia-Fuentes et al. (2009)
	Salted method	Bone mineral matrix			Pore size can be controlled by the shape of the pore-forming agent	Low pore penetration	Sommer et al. (2017)
	Porogen leaching (modified)				Pore distribution is uniform Interconnected porous structures High porosity		Chen et al. (2019)
Conduit	Mold casting/Dipping				Good shape	Brittle Poor mechanical performance	Yang et al. (2009), Ghaznavi et al. (2011)
	Electrospinning				Appropriate tensile strength	Fragile	Wang et al. (2015)
Nanoparticles	Desolvation	VEGF			Relatively mild conditions Particle size is small The operation is simple	Residual organic solvent	Kundu et al. (2010)
	Salt leaching	Model drug			No need to add a stabilizer and a crosslinking agent It has good security	Poor pelletization Weak acid drugs are not suitable	Lammel et al. (2010)
	Supercritical fluid technologies	Indomethacin			No residual organic solvent	High cost and high requirement for equipment (high voltage) Complex operation	Zhao et al. (2012)
Microneedles	Mold casting	Melatonin		Inserted into the skin	It is non-invasive and can be administered via the skin	Microneedles are liable to break and remain in the skin causing allergic reactions	Qi et al. (2021)

## 3 The application of SF-based drug delivery materials in neurological diseases

**FIGURE 3:**
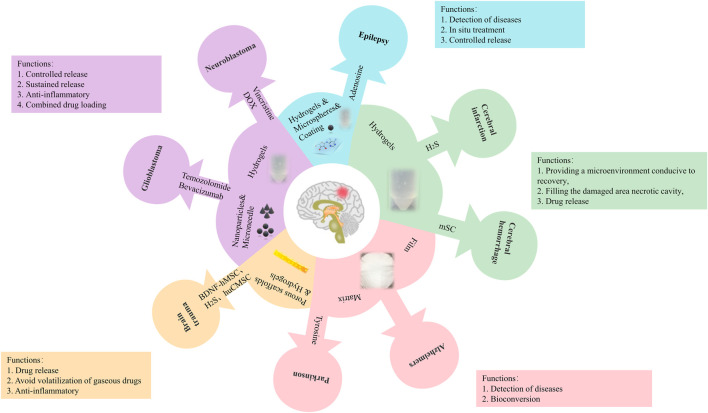
The application of SF-based drug delivery materials in neurological diseases.

**FIGURE 4:**
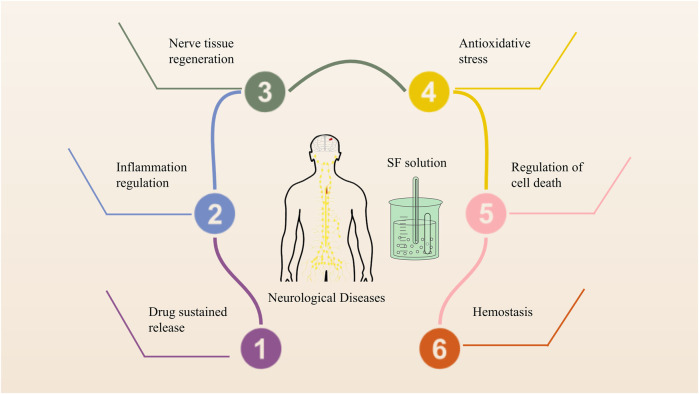
The possible molecular mechanism of SF-based drug delivery materials in neurological diseases.

There are significant socioeconomic implications for sufferers and society as a whole from neurological diseases. The penetration of drugs into the brain has been achieved using a variety of strategies (Islam et al., 2020). A variety of drug delivery systems and drug delivery vehicles have been reported to be feasible to SF (Wani and Veerabhadrappa, 2018). Previous studies have confirmed that SF showed good biocompatibility with nerve tissues and cells, which may be used to prepare nerve conduits or drug delivery vectors to treat neurological diseases (Yang et al., 2007; Tang et al., 2009). Therefore, we have summarized the applications of SF-based drug delivery materials (Figure 3) and the possible molecular mechanism (Figure 4) in neurological diseases.

In terms of sustained release systems, SF is a new option. As the core material, it is possible to design SF into many forms, including microspheres, hydrogels, films, scaffolds, nanoparticles, and microneedles and widely used in drugs or cells delivery. This process that has resulted in SF being used as a carrier for neurological diseases by laying the basic principles, such as traumatic brain injury, intracerebral hemorrhage, ischemic stroke, Alzheimer’s disease, Parkinson’s disease, glioblastoma, neuroblastoma, and epilepsy. The functions of SF as a carrier in neurological diseases include controlled and sustained drug release, filling the damaged cavity, avoiding volatilization of gaseous drugs, and providing a microenvironment for recovery.

SF has been widely used as a drug-carrier matrix and provides different protection mechanisms according to the characteristics of silk fibroin and the contained drug. SF has been widely used for the preparation of sustained drug delivery systems. SF-based drug delivery materials also regulate cellular homeostasis, such as hemostasis and nerve regeneration, and act as prime modulators of cellular dysfunction, such as inflammation, oxidative stress, cell death, and nerve regeneration, contributing to treat neurological diseases.

### 3.1 Vascular diseases

SF hydrogels bring drugs into the site of neuronal damage in the brain (targeted administration, and provides the microenvironment for stroke recovery to realize treatment. Stroke, including ischemic and hemorrhagic strokes, is one of the causes of neuron damage in the human body, and the regeneration capacity of neurons is very limited. Current strategies aimed at enhancing brain tissue’s limited capacity for spontaneous regeneration, include using biologics, small molecules, and cells to treat diseases and SF has been used as an effective delivery vector. Noh et al. reported that SF loaded with Brain Factor-7 peptide reduces brain damage and improves neurological function after stroke by reducing the generation of ROS (Noh et al., 2020a). Gorenkova et al. reported that self-assembled SF hydrogels filled the stroke cavity conducive to stroke recovery and there was no significant microglia/macrophage reaction (Gorenkova et al., 2019). Sun et al. reported that a 3D scaffold made of IKVAV-modified SF hydrogels may be useful for the differentiation and maturity of neuronal stem cells (Sun et al., 2017). Fernández-García et al. found that SF hydrogel-loaded with bone marrow mesenchymal stem cells (MSCs-SF) was better to implant into an infarcted hemisphere than SF hydrogels without MSCs, suggesting that SF is an effective delivery vector for stem cells in stroke (Fernández-García et al., 2018). Our group has recently prepared injectable SF hydrogels loaded with hydrogen sulfide (H_2_S@SF) for the treatment of severe intracerebral hemorrhage. The result showed that the neuroprotective effect of H_2_S@SF hydrogels on intracerebral hemorrhage was superior to that of SF hydrogels alone because of sustained H_2_S release and localized targeted delivery from H_2_S@SF hydrogels (Zhang et al., 2022). In general, it is possible to use SF-based drugs for the development of brain tissue engineering in stroke patients.

### 3.2 Neurodegenerative diseases

At present, the application of SF in AD is the detection and diagnosis of the disease. SF can also be used for the treatment of Parkinson’s disease, and using SF to deliver drugs is simpler and more effective than the traditional treatment of Parkinson’s disease. Alzheimer’s disease (AD) is pathologically defined by the presence of neurofibrillary tangles and the accumulation of amyloid-beta (Aβ) in the brain (Hardy and Higgins, 1992). Goncalves et al. developed a new prototype of an electrochemical immunosensor with aminoacid-long amyloid-β peptides fixed in layer-by-layer (LbL) films containing SF to make a preliminary diagnosis of AD (Gonçalves et al., 2016). Liu et al. developed an immune electrochemical interface of conductive SF-based immunoparticles (CSIPs) by electrophoretic/electropolymerization strategy that can be easily integrated into point-of-care testing (PoCT) devices. The interface comprises electropolymerized Poly 3,4-ethylene dioxythiophene (PEDOT) bridged conductive SF-based immune particles (CSIPs) to achieve the Aβ detection in the blood of patients with AD (Liu et al., 2018). There is no valid data to support the application of SF-loaded drugs in the treatment of AD, and as a result, SF-based materials can be used in treating AD in a much broader range of applications.

Parkinson’s disease (PD) is characterized by the progressive loss of dopaminergic neurons of the substantia nigra pars compacta that project to the striatum, which translates into reduced dopamine levels. Levodopa, a precursor of dopamine, is the most common method for increasing dopamine concentration in PD patients (Hayes et al., 2019). Acharya et al. devised a new method for converting tyrosine to L-DOPA that is cheaper and more efficient. By fixing tyrosinase to SF, they found the optimal conditions for L-DOPA production and also showed greater stability, allowing mass production (Acharya et al., 2008). However, it is urgent to fill the gaps in the application of SF-loaded drugs in the treatment of PD.

### 3.3 Traumatic brain injury

After SF hydrogels and porous scaffolds are injected or implanted into the site of traumatic brain injury, it can play its anti-inflammatory effect and the role of related drugs to promote the regeneration and differentiation of damaged neurons to achieve the purpose of treatment. Sultan et al. showed that human MSCs with brain-derived neurotrophic factor (BDNF) can be encapsulated with SF-based hydrogels and BDNF-hMSC was transplanted into rat brain to treat traumatic brain injury (TBI), which represents a critical setup towards brain injury treatment for clinical application. Encapsulated BDNF-HMSC induces neurogenesis by secreting BDNF *in vitro* cell culture and contributes to the growth and differentiation of neurons at the injured site by eliminating oxidative stress *in vivo* TBI model (Sultan et al., 2021). Jiang et al. fabricated porous scaffolds using cross-linked collagen and SF and co-cultured them with human umbilical cord MSCs (hUCMSCs) to form implantable complexes. The SF implant complex is injected directly into the site of injury in a TBI canine model. The result showed that the porous SF scaffolds loaded with hUCMSCs could suppress inflammation, inhibit glial proliferation, promote vascular regeneration, induce neuronal cytoskeleton and axon regeneration, and enhance synapses and myelination (Jiang et al., 2021). Our group has recently prepared injectable SF hydrogels loaded with hydrogen sulfide (H_2_S@SF) to treat TBI and surfaces filled with H_2_S@SF hydrogels can achieve local administration and sustained-release H_2_S, avoiding volatile and toxic side effects. Moreover, surfaces filled with H_2_S@SF hydrogels are capable of penetrating the blood-brain barrier, enabling non-invasive local delivery of H_2_S after brain injury (Chen et al., 2022b). SF can be formulated into various material formats and different forms of SF carriers, as promising drug delivery systems, should be further studied in the future.

### 3.4 Brain tumors

Using SF microneedles or hydrogels can achieve targeted drug delivery, control drug release, and improve drug utilization rate (compared with traditional drug delivery), to achieve the purpose of treating nervous system tumors. Ribeiro et al. reported that SF hydrogels were able to suppress the growth of human neuronal glioblastoma (U251) in response to conformational transitions from the unordered coil to the β-sheet structure, suggesting that SF is a very useful tool in specific drug delivery to target glioblastoma (Ribeiro et al., 2018). Wang et al. prepared SF microneedle (SMN) patches to inhibit neovascularization, anti-angiogenesis, and induction of tumor cell apoptosis by remoting activation and controlling the release of Bevacizumab, thrombin, and temozolomide (Wang et al., 2022). SF microneedles provide a vehicle for drug release in the desired manner. Pandey et al. constructed SF nanoparticles loaded with DOX and the DOX-SF nanoparticles induced the exogenous apoptosis pathway of glioblastoma cell lines, C-6 and LN-229 (Pandey et al., 2020). Thermal tumor ablation can be achieved through photothermal therapy using a light absorber under near-infrared (NIR) irradiation. Nevertheless, due to leakage of light absorbent, near-infrared irradiation may damage adjacent tissues. To reduce this damage, Yao et al. developed a bioinspired green hydrogel (BVSF) integrating bioproduct biliverdin to stimulate healing wounds and produce angiogenesis by controllable NIR irradiation Yao et al., 2020. An indocyanine green-SF nanoparticle platform (ICG-SFNPs) for photo-thermal treatment of glioblastoma has been developed by Xu et al. It was effective at accumulating inside tumors in mice bearing nude C6 gliomas when ICG-SFNPs were injected into their veins (Xu et al., 2018; ZhuGe et al., 2019). Coburn et al. found that treated with SF hydrogels containing a continuous release of vincristine and doxorubicin significantly reduced the growth of neuroblastoma tumors. Vincristine delivery significantly slows tumor growth and increases the availability of oncology drugs. In addition, intratumor drug concentration was significantly higher when the hydrogel was injected into the tumor site than when intravenous (Coburn et al., 2017). Therefore, these results provide a powerful tool for the investigation of different forms of SF carriers on the programmed cell death in tumors.

### 3.5 Epilepsy

Adenosine-carrying SF microspheres, nanofilm coatings, microneedles, and hydrogels can not only treat epilepsy but also monitor the delivery of drugs. Continual monitoring for symptoms and immediate *in situ* treatment for seizures are crucial for patients with epilepsy, chronic and non-communicable brain diseases affecting people of all ages. Wilz et al. developed a novel adenosine-release system based on SF by insetting adenosine-containing microspheres into a nanomembrane-coated SF scaffolds and were prepared. In this study, it was found that kindling acquisition occurs in a dose-dependent manner when treated with adenosine-releasing brain implants with the desired target release dose, suggesting that implant-derived adenosine might prevent epileptogenesis (Wilz et al., 2008). Zhang et al. developed sensor-equipped devices using a conductive SF hydrogel (CSFH) combination with microneedles array to treat epilepsy for real-time monitoring of the drug delivery (Zhang et al., 2020). These results, based on different SF designs, offer a rapid and sustained treatment to stop or prevent epilepsy.

Neurological diseases can also be classified as chronic or acute according to the speed of onset. Chronic injury can use various forms of SF to achieve the purpose of sustained and controlled release. Acute injury can lead to structural changes and defects. In addition to the sustained release and controlled release of the drug, it also needs to fill the damage site. Therefore, it is suitable for *in situ* administration with SF hydrogels to fill the defect site and provide a microenvironment suitable for nerve recovery after injury. The application of different forms of SF depends on the pathophysiological mechanisms of various neurological diseases (existing research) as follows (Table 2).

**TABLE 2:** The application of different SF forms depends on the pathophysiological mechanisms of various neurological diseases.

Classification of diseases				Physiopathologic mechanism	SF forms and usage methods	Function	References
Nervous system diseases	Acute	Vascular nature (stroke)	Ischemic strokes	Local brain tissue ischemia and necrosis occur based on atherosclerosis, forming a cavity	Hydrogels (Fill, Inject)	Local targeted drug delivery; filling the stroke cavity (caused by damage); providing a suitable microenvironment; removing ROS	Sun et al. (2017), Fernández-García et al. (2018), Gorenkova et al. (2019), Noh et al. (2020b)
			Hemorrhagic strokes	Blood vessels rupture to form a hematoma, producing emptiness, nerve damage, and inflammation			Zhang et al. (2022)
		Traumatic	TBI	Primary and secondary injury lead to long-term neuroinflammation with massive neuronal death	Hydrogels and porous scaffolds (Inject or Implant)	Filling the site of injury; inhibiting neuroinflammation and glial cell proliferation, promoting vascular regeneration, inducing neuronal cytoskeleton and axonal regeneration, and enhancing synapses and myelination	Jiang et al. (2021), Sultan et al. (2021), Chen et al. (2022a)
			SCI				
		Inflammatory	Meningitis	Infection	No reports		
			Myelitis				
	Chronic	Degenerative disease	Alzheimer’s disease	brain atrophy, neuroinflammatory plaques, neurofibrillary tangles, reduced neurons, and A β deposition	SF films, SF particles (Testing and diagnosis)	SF films binding amyloid-β 1-40 peptide can detect specific antibodies to diagnose Alzheimer’s disease	Gonçalves et al. (2016), Liu et al. (2018)
			Parkinson	Decrease of dopamine levels	SF matrix	Glutaraldehyde cross-links SF with tyrosinase, promoting the conversion of tyrosinase into dopamine	Acharya et al. (2008)
		Tumor	Glioblastoma		SF hydrogels (*In situ* injection)	Targeted drug administration, reducing ROS, good stability, and sustained release	Coburn et al. (2017), Ribeiro et al. (2018), Yao et al. (2020)
					SF microneedle patch (stick)		Pandey et al. (2020), Wang et al. (2022)
					SF nanoparticles (vein injection)		Xu et al. (2018), ZhuGe et al. (2019)
			Neuroblastoma		SF nanoparticles (injection)	Local, long-term sustained drug delivery	Montalbán et al. (2018)
					Electrospun SF mats (implantation)	Enhance cell adhesion, promoting directional growth of neurite, reducing ROS	Nune et al. (2019), Phamornnak et al. (2022)
					SF foams (implantation)	Sustained drug release, local delivery	Ornell et al. (2020), Ornell et al. (2021)
		Epilepsy	Epilepsy	Abnormal, paroxysmal electrical discharge	SF microspheres, SF coating (implantation)	Delivery drug	Wilz et al. (2008)
					SF hydrogels, microneedles (stick)	Delivery and monitoring of drug delivery	Zhang et al. (2020)

## 4 Conclusion

In conclusion, the safety and tolerability of SF as a drug carrier implanted or injected into the human body have been confirmed, indicating the feasibility of SF in the treatment of brain diseases and spinal cord injury (Chung et al., 2014; Wang et al., 2016b; Fernández-García et al., 2016; Li et al., 2016; Jiao et al., 2017; Jiang et al., 2020; Jiang et al., 2021; Wang et al., 2021; Liu et al., 2022b). Due to its biodegradable, biocompatible, mechanically strong characteristic, and better cell adhesion ability (Shen et al., 2022), SF shows great potential for sustained delivery applications of drugs and genes. SF is a versatile material that can be made into a wide range of forms, like microspheres, hydrogels, films, scaffolds, nanoparticles, and microneedles for drug delivery, which have triggered and expanded the use of SF as a carrier of neurological disorders. Biocompatible and biodegradable silk-derived materials can be used to treat neurological diseases if we can improve silk morphologies and the effects of loaded drugs to meet the needs of specific diseases. SF alleviates ischemic injury, protects nerve cells, and improves memory deficits by reducing ROS production (Noh et al., 2020b). The SF hydrogel was injected into the site of ischemic brain injury to fill the site, showing good biocompatibility and providing a good microenvironment for nerve cell regeneration (Gorenkova et al., 2019). Silk fibroin protein has good cell adhesion (Shen et al., 2022). Nevertheless, the treatment effect of a single SF on stroke is limited (Zhang et al., 2022). Therefore, more and more people use SF as a drug delivery system and obtain various forms of modified SF through various methods, or composite materials prepared with SF and other materials to improve the therapeutic effect of nervous system diseases (Sun et al., 2017). In addition, it has been found in the treatment of traumatic brain injury that SF hydrogels carry drugs that can penetrate the blood-brain barrier (Chen et al., 2022a) and inhibit glial proliferation and promote vascular regeneration (Jiang et al., 2021). SF has shown excellent drug targeting and controlled release in the treatment of nervous system tumors (Coburn et al., 2017; Yao et al., 2020). The drug delivery system and sensing device formed by the preparation of SF can control drug release and monitor drug delivery, effectively preventing and treating epilepsy (Wilz et al., 2008; Zhang et al., 2020). SF can also be used as an immune sensing system to detect Aβ levels in the blood to diagnose Alzheimer’s disease (Hardy and Higgins, 1992; Gonçalves et al., 2016; Liu et al., 2018). In addition to its applications in brain diseases, SF has also played a role in the treatment of spinal cord injuries. Liu et al. prepared a hydrogel that can be formed *in situ* for the treatment of spinal cord injury. The hydrogel is biocompatible, stretchable, mechanically matched, and adhesive to the primary spinal cord. At the same time, the hydrogel fills in the damaged area, promoting nerve regeneration and, ultimately, functional recovery (Liu et al., 2022b). As with brain diseases, the treatment of spinal cord injury uses SF to deliver NSCs (Jiang et al., 2020) and neurotrophic factors (Li et al., 2018) to promote nerve regeneration after spinal cord injury. Now, it has been found that human olfactory ecto-mesenchymal stem cells (hOE-MSCs) can differentiate into motor neurons and promote the recovery of motor function after spinal cord injury, which provides a new promising direction for clinical stem cell therapy (Hamidabadi et al., 2021). However, SF-based materials are still in their infancy, and many aspects require further development and improvement before they can be used to treat most neurological diseases. Alginate-magnetic short nanofibers 3D composite hydrogel enhances the biological activity of hOE-MSCs (Karimi et al., 2021). Therefore, we considered whether we could make a composite of SF together with alginate, magnetic short nanofibers or polycaprolactone (PCL), and poly lactide-co-glycolic acid (PLGA) (Zare et al., 2021). It can not only make the material have good biocompatibility, and optimize the mechanical properties of the material, but also enhance the activity of the cells transported by the material, to promote nerve regeneration after injury. In a word, fabricating various forms of SF-based biomaterials promising approaches to achieve sustained release of drugs and genes in neurological diseases.

## Figures and Tables

**Table:** 

**SF**	Silk fibroin
**PLGA**	Polylactic co-glycolic acid
**hMSCs**	Human bone marrow-derived mesenchymal stem cells
**PEG**	Polyethylene glycol
**BMP-2**	Bone morphogenetic protein-2
**rhBMP-2**	Recombinant human bone morphogenetic protein-2
**IGF-1**	Insulin-like growth factor 1
**rhIGF-1**	Recombinant human insulin-like growth factor 1
**DOX**	Doxorubicin
**CMCS**	Carboxymethyl chitosan
**TA**	Tannic acid
**CSMA**	Methacrylated chitosan
**SFMA**	Methacrylated SF
**NPs**	Nanoparticles
**HAP**	Hydroxyapatite
**PVA/SF/N-HA**	Porous polyvinyl alcohol/SF/nano-hydroxyapatite
**GP**	Genipin
**HCFs**	Human corneal fibroblasts
**PBS**	Phosphate buffer solution
**SCMK**	S-carboxymethyl keratin
**rBMSC**	Rat bone marrow-derived mesenchymal stem cells
**DBM**	Demineralized bone matrix
**SC-CO2**	Supercritical Carbon Dioxide
**AB**	Ammonium bicarbonate
**NC**	Nerve catheters
**NCs**	Nerve conduits
**NGC**	Nerve guidance conduit
**ESP-NGCs**	NGCs made from silk fibroin and poly(lactic-co-glycolic acid) through electrospinning and weaving
**DMSO**	Dimethyl sulphoxide; VEGF, vascular endothelial growth factor
**SEDS**	Solution-enhanced dispersion by supercritical CO2
**IDMC**	Indomethacin
**MNs**	Microneedles
**SFS**	Straining flow spinning
**MI**	Myocardial infarction
**NGF**	Nerve growth factor
**BDNF**	Brain-derived neurotrophic factor
**MCAO**	Middle cerebral artery occlusion
**mSC**	Mesenchymal stem cells
**Aβ**	Amyloid-beta
**AD**	Alzheimer’s disease
**LbL**	Layer-by-layer
**Aβ1-40**	The 40 aminoacid-long amyloid-β peptides
**CSIPs**	Conductive SF-based immunoparticles
**PoCT**	Point-of-care testing
**PEDOT**	Poly(3,4-ethylene dioxythiophene)
**CSIPs**	Conductive silk fibroin-based immunoparticles
**PD**	Parkinson’s disease
**L-DOPA**	3,4-dihydroxyphenyl-L-alanine
**TBI**	Traumatic brain injury
**BDNF-hMSC**	Encapsulate hMSCs to overproduce BDNF
**TBI**	Traumatic brain injury
**hUCMSCs**	Human umbilical cord mesenchymal stem cells
**GBM**	Glioblastoma
**SMN**	SF protein microneedle
**NIR**	Near-infrared
**BVSF**	Bioinspired green hydrogel
**NSCs**	Neural stem cells
**hOE-MSCs**	Human olfactory ecto-mesenchymal stem cells
